# Supplementary material for: Hammerhead flatworms (Platyhelminthes, Geoplanidae, Bipaliinae): mitochondrial genomes and description of two new species from France, Italy, and Mayotte
Source: PeerJ. 2022 Feb 1;10:e12725. doi: 10.7717/peerj.12725 (PMC8815365; doi:10.7717/peerj.12725)
Supplement: Supplemental Information 1 [file peerj-10-12725-s001.pdf]

Supplemental File

Specimens of *Humbertium covidum* examined for the present study.

A total of 30 specimens were examined.

Geographic origin of specimens :

SP: Saint-Pée-sur-Nivelle, Pyrénées Atlantiques, France

BI: Billère, Pyrénées Atlantiques, France

CA: Casier, Treviso, Italy.

In addition, several specimens from Billère (similar to JL343), and several specimens from Casier (similar to JL351) were shipped to Pierre Gros, photographed alive by him, then destroyed. A specimen (similar to JL351) was filmed alive *in situ* by Enrico Ruzzier.

Specimen	Locality	Status	State	cox1	28S	18S	Mitogenome	Slides
JL090A	SP	Voucher	Damaged (mol.)	MG655588				
JL090B	SP	Voucher	Destroyed	MZ561471 *	MZ520989	MZ520996	MZ561471	
JL090C	SP	Voucher	Untouched					
JL090D	SP	Voucher	Untouched					
JL090E	SP	Voucher	Untouched					
JL090F	SP	Voucher	Untouched					
JL343	BI	Voucher	Damaged (mol.)	MZ622153				
JL351A	CA	Paratype	Damaged (hist.)					34
JL351B	CA	Holotype	Damaged (hist.)					36
JL351C	CA	Paratype	Untouched					
JL351D	CA	Paratype	Untouched					
JL351E	CA	Paratype	Untouched					
JL351F	CA	Paratype	Untouched					
JL351G	CA	Paratype	Untouched					
JL351H	CA	Paratype	Damaged (mol.)	MZ622148				
JL351J	CA	Paratype	Damaged (mol.)	MZ622149				
JL351K	CA	Paratype	Damaged (mol.)	MZ622150	MZ647546			
JL351L	CA	Paratype	Damaged (mol.)	MZ622151	MZ647547			
JL351M	CA	Paratype	Damaged (mol.)	MZ622152	MZ647548			
JL351N	CA	Paratype	Destroyed	MZ561472 *	MZ520988	MZ520995	MZ561472	
JL351P	CA	Paratype	Destroyed					
JL351Q	CA	Paratype	Untouched					
JL351R	CA	Paratype	Untouched					
JL351S	CA	Paratype	Untouched					
JL351T	CA	Paratype	Untouched					
JL351U	CA	Paratype	Untouched					
JL351V	CA	Paratype	Untouched					
JL351W	CA	Paratype	Untouched					
JL351X	CA	Paratype	Untouched					
JL351Y	CA	Paratype	Untouched					

* a reminder that the *cox1* sequence is included in the corresponding mitogenome sequence

Damaged (mol.): a small part (about $1mm^3$) was taken from the specimen.

Damaged (hist.): the specimen was processed for histology.

Destroyed: the whole specimen was used for molecular analysis.

Specimens of *Diversibipalium mayottensis* examined for the present study.

A total of 7 specimens.

All specimens collected by Laurent Charles in Mayotte.

In addition, one photograph was received of another specimen, not collected (see Justine et al., 2018, Figure 22, photo by Benoît Duperron).

Specimen	Locality	Status	State	cox1	28S	18S	Mitogenome	Photos
JL282	Ouangani	Holotype	Damaged (mol.)	MG655598				Figures 24-25
JL280	Mtsamboro	Paratype	Regenerating + damaged (mol.)	MG655596				Figures 26-27
JL281A	Mtsamboro	Paratype	Highly damaged (mol.)	MG655597 § MZ561470 *	MZ520986	MZ520997	MZ561470	
JL281B	Mtsamboro	Paratype	Damaged (mol.)	MG655597 §				
JL281C	Mtsamboro	Paratype	Damaged (mol.)	MG655597 §				
JL283	Mamoudzou	Paratype	No head visible					
JL284	Mtsamboro	Paratype	Damaged (mol.)	MG655599				

* a reminder that the *cox1* sequence is included in corresponding mitogenome sequence

§ sequence MG655597: one sequence deposited in GenBank, based on 3 identical replicates from specimens JL281A, JL281B, JL281C

Damaged (mol.) : a small part (about 1mm^3) was taken from the specimen.

Highly damaged (mol.): most of body destroyed, only head remaining.
